# Injuries in children and adolescents with psychiatric disorders

**DOI:** 10.1186/s12889-020-09283-3

**Published:** 2020-08-24

**Authors:** Sara Agnafors, Jarl Torgerson, Marie Rusner, Anna Norman Kjellström

**Affiliations:** 1 Division of Children’s and Women’s health, BKV, Department of Biomedical and Clinical Sciences, Linköping University, S-581 85 Linköping, Sweden; 2 Department of Research, Södra Älvsborgs Hospital, Borås, Sweden; 3 Department of Psychosis, Sahlgrenska University Hospital, Gothenburg, Sweden; 4 Institute of Health and Care Sciences, Sahlgrenska Academy, University of Gothenburg, Gothenburg, Sweden; 5 Department of Data Management and Analysis, Head Office, Region Västra Götaland, Skövde, Sweden

**Keywords:** Mental health, Injuries, Maltreatment, Children

## Abstract

**Background:**

Unintentional injuries are a leading cause of morbidity and mortality in children of all ages. Prevention strategies require knowledge of risk factors, and behavior and psychiatric disorders have been suggested to influence the risk of injury during childhood. While externalizing disorders have been found to increase the risk for injuries, results are mixed regarding internalizing disorders, such as affective and anxiety conditions, and Autism Spectrum Disorders (ASD). There is a need for large scale studies relying on robust data sources. The aim of the present study was to examine the association between psychiatric disorders and injuries requiring medical attention, in a large population-based cohort of 350,000 children and adolescents in Sweden.

**Methods:**

Data were obtained from the regional health care database Vega. Psychiatric diagnoses and injury diagnoses obtained during 2014–2018 for individuals aged 0–17 years in 2016 were extracted. Descriptive statistics were used to examine differences in 5-year injury prevalence between children with and without different psychiatric diagnoses. Logistic regression was used in age-stratified models to test the association between psychiatric diagnoses and injuries requiring medical attention.

**Results:**

The results show an increased risk for concurrent injuries in general, but the patterns vary by age and psychiatric disorder. Externalizing disorders and anxiety conditions were associated with concurrent injuries, while individuals with ASD had a lower risk for most injuries included. Affective disorders were associated with an increased risk for wounds, concussion, complications and poisoning, while the risk for fractures was decreased. Self-inflicted injury was more common in all psychiatric conditions investigated during adolescence, except for ASD. Children and adolescents with many types of psychiatric disorders were also at increased risk for a concurrent maltreatment diagnosis.

**Conclusions:**

A general pattern of increased risk for concurrent injuries in children and adolescents with most psychiatric diagnoses was found, but the associations vary by age and type of psychiatric disorder. The results add to the literature on risk factors for injuries in children and adolescents, supporting diagnosis specific patterns. Several psychiatric diagnoses were associated with a marked increase in injury risk, indicating a high burden of disease for affected individuals.

## Background

Unintentional injuries are a leading cause of morbidity and mortality in children of all ages [1]. Besides the immediate consequences for the child and its family, unintentional injuries constitute a major impact on health care and society in terms of hospitalization, emergency care visits and residual disabilities. Prevention strategies requires identification of at-risk groups. There is an unequal distribution in injury prevalence related to socioeconomic status (SES) [1, 2] but also with respect to gender [2, 3]. Moreover, child behavior and associated psychiatric disorders have been brought to light as risk factors for unintentional injuries in children and adolescents.

Firstly, accident proneness is included as one characteristic of Conduct Disorder (CD) and Attention Deficit Hyperactivity Disorder (ADHD) [4]. In a recent meta-analysis, increased injury prevalence was found among children with ADHD [5]. The core symptoms of ADHD (inattention, hyperactivity and impulsivity) are predisposing for behaviors that could increase the risk for injuries. Smaller studies have indicated that children with ADHD are less likely to perceive consequences of hazardous situations [6] and that impulsivity as a trait is associated with risk behaviors such as initiating physical fights [7] which in turn could increase the risk for injuries. However, other studies found no association between ADHD and injuries after controlling for comorbidity [8]. In contrast, in a European community study, children expressing symptoms of oppositional defiant disorder (ODD) were at increased risk of past-year injury compared to their peers [9]. In a large population-based cohort, Rowe et al. found ODD to be associated with unintentional injuries after controlling for comorbid psychopathology, while no association was found for conduct disorder (CD) [10]. In a clinical sample, however, conduct problems were shown to be associated with hospital admission due to unintentional injury [11].

While the association between injuries and externalizing disorders has been addressed in population-based as well as clinical samples, less is known about injury prevalence in children with internalizing disorders such as depression and anxiety. Results on the association between anxiety disorders and the risk of injury have been mixed. A population-based study on pre-school children found no association between either anxiety or depression and injuries [12]. In a twin study, Rowe et al. (2007) found an association between anxiety and parent reported injury during the preceding 3 months [13]. In contrast, Jokela et al. 2009 noted a decreased risk for injury in adolescence and adulthood for individuals with internalizing symptoms at ages 7 and 11 [14]. The latter study, however, relied on teacher reports on internalizing problems and parents- and self-reports on injury incidence. Depressive symptoms have been shown to be associated with increased risk for injuries in adolescence using cross sectional self-reported data [15].

The literature on the association between autism spectrum disorder (ASD) and unintentional injuries is limited. In a population-based study on adolescents, the risk for injury during the last 12 months was decreased in individuals with autism spectrum disorders [16]. In another study by Bonander et al., children aged 6–17 with ASD were not found to have an increased risk for injuries [17]. Similar results have been found in smaller samples [18].

Likewise, children and adolescents with psychiatric disorders may be at increased risk for *intentional* injuries, such as maltreatment and interpersonal violence. A population-based register study found children with mental and behavioral disorders to be at increased risk for maltreatment while no significant associations were found between ASD and maltreatment after controlling for child, family and neighborhood risk factors [19]. Several studies have confirmed an association between different psychiatric diagnoses and maltreatment [20, 21], however, the association seem to be more complex than childhood maltreatment causing mental health problems [22].

Self-injury is associated with many types of psychiatric disorders, such as depression, anxiety, eating disorders, ADHD, CD and ODD [23–27]. Moreover, non-suicidal self-injury (NSSI) has been found to be associated with impulsivity [24], which, in turn, also increases the risk for unintentional injuries [13]. In the present study, self-injury was used as a control variable. This was done in order to limit the risk for higher prevalence of injuries that could be self-inflicted (for example wounds or poisoning) in individuals with psychiatric disorders. Self-injury was also used as an outcome variable investigating comorbidity with different psychiatric disorders.

Previous studies have been criticized for using poor measures including retrospective reports. Moreover, the diverging characters of different psychiatric disorders call for disorder specific analyses, controlling for psychiatric comorbidity. Thus, there is a need for large scale studies relying on robust data sources.

The aim of the present study was to examine the association between psychiatric disorders and injuries requiring medical attention, in a large population-based cohort of 359,597 children and adolescents in western Sweden. The study adds to the existing data by 1) the use of a large population-based cohort, 2) the use of register data excluding the risk for recall bias and enabling control for psychiatric comorbidity and 3) the inclusion of internalizing disorders which has not been as thoroughly studied as externalizing disorders.

## Methods

### Subjects

This is a cross-sectional study. All individuals aged 0–17 years, who were residents of Västra Götaland in 2016, constituted the study population. Register data on diagnoses was obtained from the regional health care database Vega. Vega holds information about date of contact, type of contact, healthcare provider, diagnoses, operations, health centers and hospitals and age and sex of the patient. Diagnoses included in Vega are coded according to the International Statistical Classification of Diseases and Related Health Problems 10th revision, ICD-10. Public as well as private care givers are obliged to deliver data to Vega, and the register thus contains information about all primary and specialist health care in Region Västra Götaland, Sweden.

### Measures

Information on psychiatric diagnoses and injuries requiring health care during the years 2014–2018 as defined in Table 1, were obtained from Vega for all individuals aged 0–17 years in 2016 (*n* = 324,157). External causes related to injury were also obtained. Using data from Statistics Sweden, the study population was then completed with individuals aged 0–17 in region Västra Götaland in 2016 without these diagnoses based on age and gender. This resulted in a study population of *n* = 363,554. Individuals were divided into three categories based on age in 2016; 0–6 years, 7–12 years, and 13–17 years.

**Table 1:** Classification of psychiatric diagnoses, injuries and external causes

**Psychiatric diagnosis**	ICD-10 code	***n***
Schizophrenia, schizotypal and delusional disorders	F20-F29	116
Affective disorders	F30-F39	7560
Neurotic, stress-related and somatoform disorders	F40-F48	17,449
Autism	F84.0, F84.1, F84.5, F84.9	5079
ADHD	F90	10,497
CD, ODD	F91	1152
**Injury**		
Fractures	S02, S12, S22, S32, S42, S52, S62, S72, S82, S92, T02	32,107
Wounds	S01, S11, S21, S31, S41, S51, S61, S71, S81, S91, T01	38,054
Concussion	S06	7211
Complications	T80-T88	2094
Poisoning	T36-T50, T96	1263
Maltreatment and abuse	T74	1213
Self-inflicted injury	X60-X84, Z915	1033
**External cause**		
Falls	W00-W19	50,523
Traffic accidents	V01-V79	6798
Transport accidents	V80-V99	1586

Note: *ICD* International Statistical Classification of Diseases and Related Health Problems 10th revision

The psychiatric diagnoses investigated were selected to cover common childhood psychiatric conditions. Injury diagnoses and external causes were chosen based on previous studies and prevalence. Subjects with developmental delays (ICD-10 F82-F83, F84.2-F84.4, F84.8, F88-F89) were excluded from the study population, since these conditions could be associated with psychiatric and somatic conditions as well as gross motor delay, and thus potentially bias the results. This resulted in a total number of 359,597, and out of these, 40,579 had a psychiatric diagnosis. For detailed information on ICD codes and categorization of psychiatric diagnoses and injury types, see Table 1.

### Data analysis

First, frequencies of psychiatric diagnoses, injuries and external causes were presented by descriptive statistics. Chi^2^ was used to examine differences in injury prevalence between children diagnosed with psychiatric disorders, and those who were not. Next, the risk for concurrent injury in children with psychiatric diagnoses was investigated by logistic regression. Individuals with psychiatric diagnoses were compared to individuals without psychiatric diagnoses (reference group). Not all categories of injuries and external causes were modelled in logistic regression due to the low number of cases (see additional file). Models that were age stratified were controlled for gender, and non-age stratified models were controlled for gender and age. All psychiatric diagnoses were entered simultaneously in the models, except for age group 0–6, where psychosis was excluded due to the low number of cases. Moderate correlations were found between anxiety and affective disorders (*r* = 0.43), and ADHD and ASD (*r* = 0.41), whereas correlations between the other independent variables were lower, indicating low degree of multicollinearity. In order to maintain the integrity of the study participants, data was not shown when analyses resulted in cases of five or fewer. A *p*-value < 0.05 (two-sided) was considered statistically significant. Results from logistic regressions are presented with corresponding Odds Ratios (OR) and 95% Confidence Intervals (CI). All analyses were conducted using SPSS version 24 (IBM Corporation, Armonk, NY).

## Results

### Descriptives

#### Unintentional injuries

Amongst children with psychiatric diagnoses, 33.9% were diagnosed with an injury during the five-year period compared to 30.6% of children without a psychiatric diagnosis (χ^2^ 184.181, *p* < 0.001). In general, unintentional injuries were more common in children and adolescents with psychiatric disorders than those without, with the biggest difference noted for concussion (see additional file). In diagnosis specific analyses, injuries were more common for all psychiatric conditions except affective disorders and ASD. Large differences were noted for ODD/CD where 45.4% of 13–17-year-olds with an ODD or CD diagnosis had sought medical attention for an injury compared to 27.1% without these diagnoses. On the other hand, wounds were less common in children and adolescents with anxiety or affective disorders. Self-inflicted injury was more common for all psychiatric diagnoses from age 7 and above. For some diagnoses, differences varied by age. For example, fractures were more common in children up to age 12 with anxiety than in those without, however between age 13–17 fractures were slightly less common in adolescents with an anxiety diagnosis. For individuals with ASD, prevalence rates of fractures, wounds and concussion were very similar to that of the general population and no significant differences were found. Complications stood out as more common in individuals with ASD; 1.1% compared to 0.6% in individuals without an autism spectrum disorder. For psychotic conditions, there were mostly too few cases (*n* = 116) to run analyses. Injuries in general were not more common in children and adolescents with psychotic conditions. The only significant difference was noted for injury related to foreign body, however this result needs to be interpreted with caution due to the small number of cases.

#### Intentional injuries

For injuries not classified as unintentional, maltreatment stood out with a five-year prevalence of 1.5% in children and adolescents with psychiatric diagnoses compared to 0.2% in individuals without (see additional file). Diagnosis specific analyses revealed the same pattern, with a five-year prevalence of maltreatment diagnoses ranging from 1.7% for ADHD to 2.5% for children with anxiety conditions. This also applied to autism spectrum disorder, where 1.0% of children with ASD had a diagnosis of maltreatment, compared to 0.3% of those without.

#### External causes

External causes such as falls, traffic accidents and transport accidents were more common in children and adolescents with psychiatric disorders (see additional file). For example, 4.1% of 0–17-year-olds with ADHD had experienced a traffic accident compared to 1.8% of individuals without an ADHD diagnosis. This pattern continued in age stratified analyses in general from age 7 and above. The exception to the rule was autism spectrum disorders, where falls were less common in individuals with ASD than in children without. Regarding other external causes, no significant differences emerged for ASD.

### Logistic regression

#### ADHD

ADHD was associated with an increased risk for unintentional injuries (fractures, wounds, and concussion) in children and adolescents age 0–17 (Table 2). The same applied to complications and poisoning. When analyses were run separately for the three different age categories, no association was found between ADHD and fractures at age 0–6 or 7–12. Self-inflicted injuries were more common in individuals with ADHD with an exception of age 7–12 (Table 4). A diagnosis of maltreatment was almost twice as common in children and adolescents with ADHD, with an exception for the youngest age category.

**Table 2:**
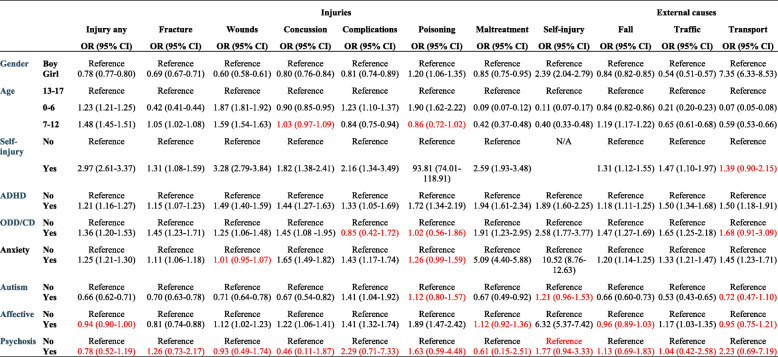
Multiple logistic regression presenting the odds ratio (OR) and corresponding 95% confidence intervals (CI) for injury and external causes in children age 0–17*

Note: * Adjusted for all variables presented in the table. *ADHD* Attention Deficit Hyperactivity Disorder, *ODD* Oppositional Defiant Disorder, *CD* Conduct Disorder. Text in red = non-significant results

An increased risk for falls and traffic accidents were found during all of childhood for individuals with ADHD, while transport accidents were more common from age 7 and above.

#### ODD and CD

ODD and CD increased the risk for unintentional injuries in adolescents, and significant associations were also found when all ages were analyzed together (Table 2). There was a marked increase in poisoning for children age 0–6 with ODD/CD, with an OR of 4.91. ODD/CD was associated with self-inflicted injury from age 7 and above. Likewise, an association was found between ODD/CD and maltreatment when all ages were analyzed together.

In the total population, an increased risk was found for falls and traffic accidents, however, the results were not consistent in the subgroups, probably due to the low count. For example, there was an eight-fold increase of transport accidents for 0–6-year-olds with a concurrent ODD or CD diagnosis (Table 3), while no significant results were found at age 7–12 or 13–17.

**Table 3:**
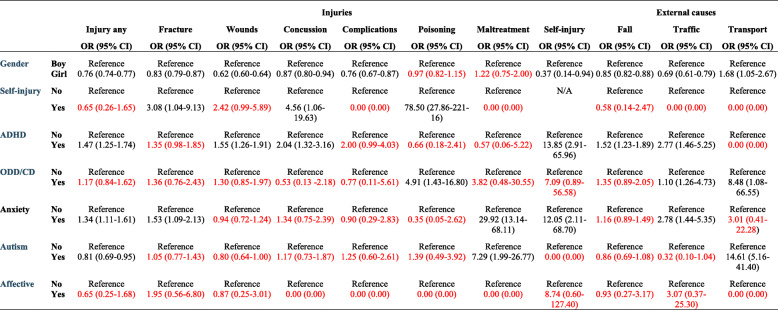
Multiple logistic regression presenting the odds ratio (OR) and corresponding 95% confidence intervals (CI) for injury and external causes in children age 0–6*

Note: * Adjusted for all variables presented in the table. *ADHD* Attention Deficit Hyperactivity Disorder, *ODD* Oppositional Defiant Disorder, *CD* Conduct Disorder

Text in red = non-significant results

#### Anxiety

Anxiety conditions were associated with an increased prevalence of injuries in general and with fractures and complications (Table 2). Concussion was more common in children and adolescents with anxiety diagnoses from age 7 and above, and in the total population. No association was found between anxiety conditions and wounds. Odds Ratios for concurrent self-inflicted injury were above 8 in all age categories. There was a considerable association between anxiety conditions and maltreatment diagnosis, with OR ranging from 4.00 in adolescents (Table 5) to 29.92 in children age 0–6 (Table 3), however cases were few.

Significant associations were found between falls, traffic accidents and transport accidents in the total population and in the two oldest age categories. Furthermore, an OR of almost 3 was found between anxiety diagnoses and traffic accidents at age 0–6 (Table 3).

#### Autism spectrum disorders

In general, autism spectrum disorders were associated with a lower prevalence of unintentional injuries (Table 2). The only exception was non-significant results for fractures, wounds and concussion in age 0–6 and an increased risk for complications in age 13-17 and when all ages were analyzed together. ASD was not associated with concurrent self-inflicted injuries. An increased risk for maltreatment was found in 0–6-year-old children with ASD, however, the low number of cases indicates uncertainty (Table 3). A decreased risk for maltreatment was noted in the total population and in adolescents.

Likewise, a significant negative association was found between autism spectrum disorders and falls and traffic accidents. There was an increased risk for transport accidents in 0–6 aged children with autism (OR 14.61, CI 5.16–41.40), a result that need to be interpreted with caution due to the wide CI.

#### Affective disorders

No significant differences were noted in injury prevalence in general between individuals with affective disorders and those without (Table 2). Wounds, concussion, complications and poisoning were more common in children and adolescents with affective disorders than in those without. A lower risk for fractures was noted (Table 2). Up to age 12, the prevalence of affective disorders was low, and no significant differences were found. Affective disorders were also associated with concurrent self-inflicted injury from age 7 and above. Diagnosed maltreatment was more common in adolescents with affective disorders, but no association was found for the two youngest age categories or in the total population.

No general pattern of associations was found for external causes. A positive association was noted for falls in age 7–12 (Table 4) and for traffic accidents in the total population and in age 13–17 (Table 5).

**Table 4:**
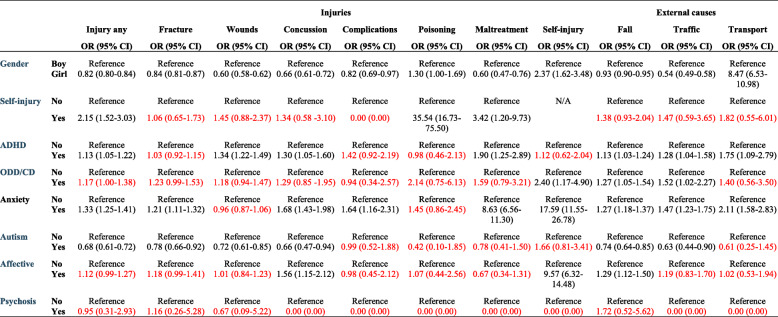
Multiple logistic regression presenting the odds ratio (OR) and corresponding 95% confidence intervals (CI) for injury and external causes in children age 7–12*

Note: * Adjusted for all variables presented in the table. *ADHD* Attention Deficit Hyperactivity Disorder, *ODD* Oppositional Defiant Disorder, *CD* Conduct Disorder

Text in red = non-significant results

**Table 5:**
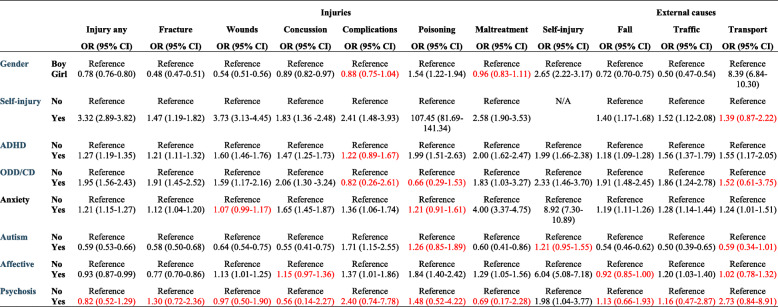
Multiple logistic regression presenting the odds ratio (OR) and corresponding 95% confidence intervals (CI) for injury and external causes in children age 13–17*

Note: * Adjusted for all variables presented in the table. *ADHD* Attention Deficit Hyperactivity Disorder, *ODD* Oppositional Defiant Disorder, *CD* Conduct Disorder

Text in red = non-significant results

#### Psychotic conditions

Psychotic conditions were associated with self-injury in adolescents. There were no associations with increased prevalence of any other type of injury or external causes.

## Discussion

The aim of the present study was to examine the association between psychiatric disorders and injuries in a large population-based cohort of 359,597 children and adolescents in western Sweden. Injury prevalence was found to vary between different psychiatric disorders.

ADHD was associated with increased injury prevalence at all ages with few exceptions. The core symptoms of inattention, concentration problems and hyperactivity could all increase the risk for injuries. Effect sizes were roughly in line with previous results [5]. Perhaps more unexpectedly, no significant association was found between ADHD and fractures below age 13. A tentative explanation might be diminished physical activity in general for children [28], but future studies are needed to further explore this finding. While it was not the scope of the present paper, information on the use of medication (i.e. central stimulants) would have been interesting to enable subgroup analyses. The percentage of children on medication for ADHD in the present population is not known, however, a recent meta-analysis found ADHD-medication to have a protective effect on the association between ADHD and unintentional injuries [5]. However, another study found that children treated with methylphenidate (MPH) sustained injuries of various types to a greater extent than children without MPH medication [29]. In sum, children and adolescents with ADHD are at increased risk for many types of injuries, and the absence of an association between ADHD and fractures in children should be studied further.

In general, all types of injuries included were more prevalent in adolescents with ODD or CD, which is in line with previous studies [9, 11]. Previous studies modelled ODD and CD separately, which, in a larger sample, might have yielded a different result. Relatively few individuals were diagnosed with ODD or CD in the present study. There is a considerable comorbidity between ODD, CD and ADHD [30], and diagnosis registration practices could possibly impact the prevalence if for example secondary diagnoses are left out. One finding that stood out, is the five-time increased risk for poisoning in children age 0–6 with ODD or CD. However, the prevalence rate was low, and future studies are needed to explore the specific risks for small children with behavioral problems.

ASD was associated with a decreased risk for concurrent unintentional injuries compared to the general population. Previous results have been mixed, indicating positive associations [18], negative associations [16] and results non-deviant from the general population [17]. Diverging results might in part be explained by the inclusion of relevant control factors. There is a well-known comorbidity between ASD and ADHD (in the present study, 61% of individuals with ASD had an ADHD diagnosis), which stresses the need to control for ADHD in analyses. An exception from the otherwise decreased risk was no significant difference of risk for concurrent fractures in 0–6-year-old children. This result stands in contrast to a study by Furlano et al. (2014), showing a lower risk for fractures in boys with ASD compared to boys without an ASD diagnosis [31]. The lower risk for musculoskeletal injuries in children with ASD has been hypothetically attributed to a lower degree of social interaction and a decreased likelihood of sports activities participation [32]. Moreover, in the present study, ASD was associated with an increased prevalence of complications. Conversely, in a study on morbidity after tonsillectomy, children with ASD were less likely to experience complications [33]. However, anesthetic procedures have been shown to differ between children with ASD and those without [34], possibly impacting postoperative recovery. Communicative difficulties, and difficulties adhering to medical ordinations could plausibly increase the risk for complications in this group. Clearly, the injury pattern for ASD stands in contrast to that of most other psychiatric disorders presumably due to behavior and activity. In sum, the results of the present study add to the literature stating a lower risk of injuries in children with ASD, after controlling for psychiatric comorbidity.

Regarding internalizing problems, the results varied by diagnosis. An increased risk for concurrent unintentional injuries were noted for anxiety conditions, with the exception of wounds. Affective disorders were associated with an increased risk for concussion, wounds, poisoning and complications. Previous studies on the association between internalizing disorders and injuries has been mixed. The mechanisms of action behind the association between internalizing disorders and unintentional injuries are perhaps somewhat less clear compared to externalizing disorders. Rowe et al. (2004) argue for plausible preoccupation with depressive or anxious cognitions that detracts attention and performance anxiety inhibiting the functioning [10]. Moreover, development of anxiety or depressive symptoms as a result of major injuries or traumatic events is common [35, 36]. A tentative explanation to the association found between affective disorders and concussion found in the present study is that depressive and cognitive symptoms are caused by head trauma, however, the cause of direction cannot be determined using cross sectional data. Longitudinal studies would be valuable to sort out the real impact of internalizing conditions on injury proneness.

Significant associations between psychiatric disorders and maltreatment was noted in the present study, however, the direction of the relationship varied by diagnosis and age. Autism was the only diagnosis associated with a decreased risk for a concurrent maltreatment diagnosis when analyzing all ages together. The positive association found between autism and maltreatment in age 0–6 might be due to the low number of cases, considering the wide CI. Previous studies have shown both negative [19] and positive [37] associations between ASD and maltreatment. The most consistent association was found for anxiety conditions, with a five-times increased risk for a concurrent maltreatment diagnosis in age 0–17. The ICD-code T74 includes many types of maltreatment – neglect, psychological, physical and sexual abuse. Thus, this category comprises both cases of repeated child abuse and cases of adolescent experience of assault, which might have different consequences for the victim. Maltreatment is, however, most likely underdiagnosed considering the use of register data. Moreover, a positive bias in relation to psychiatric diagnoses might be present, considering the contact with health care which increases the possibility of detecting and reporting maltreatment. Nevertheless, patients with many psychiatric disorders are at increased risk of maltreatment which has to be recognized in clinical settings, regardless of the direction of causation.

Self-injury was more commonly diagnosed in girls than in boys, which is in line with previous studies [38]. Anxiety was significantly associated with self-injury independent of age. Affect regulation has been described as a commonly reported function of NSSI [39], and comorbidity between NSSI and anxiety conditions have been reported previously [23]. ADHD, ODD/CD and affective disorders were also associated with self-injury diagnoses, in accordance with the literature. A significant association was found between psychotic conditions and self-injury in adolescents, even when controlling for psychiatric comorbidity. Psychotic experience (PE) has previously been shown to be associated with NSSI in children and adolescents [40], however, to our knowledge there is a lack of studies investigating the association between psychotic disorders and self-injury before adulthood. Previous studies indicate that NSSI is largely underreported [41], making conclusions drawn from register data somewhat precarious.

As expected, boys had an increased risk of injuries compared to girls, with the exception of self-inflicted injury. Regarding external causes, transport accidents were far more common in girls, explained by a large gender difference for ICD-code V80 referring to horse riding accidents. With respect to age, the injury prevalence was highest in age 7–12, with more than one in three needing medical attention due to injury during the 5-year study-period. Age differences are interesting in several aspects. In the youngest children, injury proneness is influenced not only by own behavior, but also by parental ability [42]. In adolescents, other factors such as alcohol and drug use and risk-taking behaviors could impact the occurrence of injury. Moreover, the intention might not always be clear or communicated in the emergency department, possibly resulting in cases of self-harm and suicide attempts passing undetected. In sum, age and gender impact the risk of injury and differences need to be taken into consideration in prevention and intervention strategies.

Possible mechanisms behind the association between mental health and injuries has been discussed. Rowe et al. (2007) suggest three pathways [13]. Psychopathology might lead to injury by for example risk taking behaviors, impulsivity or inattention. On the other hand, injury might induce psychopathology, the classical example being anxiety or depression following trauma [35, 36]. Moreover, shared or correlated risk factors might increase the risk for both psychopathology and injury. Regardless of the direction of the relationship, the present study shows that many psychiatric conditions are associated with an increased prevalence of unintentional injuries, intentional injuries and exposure to maltreatment. This is important knowledge for understanding the functioning and burden of disease for children and adolescents with mental health problems.

### Strengths and limitations

The study has several strengths, i.e., the use of a large population-based sample covering all ages and the use of register data eliminating the effect of recall bias. Moreover, all models were controlled for psychiatric comorbidity, enabling evaluation of the impact of each specific psychiatric diagnosis. However, the following limitations need to be taken into consideration.

First, the study outline did not enable controlling for socio-economic factors. Previous studies have shown an association between injuries and parental SES [2], and likewise, associations have been found between parental SES and mental health in children [43]. In a study by Bijur et al. (1986), associations between child behavior and accidental injuries were diminished after controlling for SES, however, the associations between aggression and overactivity and injuries remained [44]. Second, the use of cross-sectional data prevents interpretations about cause and effect. Severe accidents are likely to cause internalizing problems such as anxiety or depressive symptoms, and possibly also externalizing behaviors. Longitudinal studies are needed to further understand the association between mental health and injuries. Third, while the use of register data diminishes recall bias, it largely depends upon the accuracy of diagnosis setting practices. Non-specific injuries constituted a large proportion of the registered injuries (46%), rendering questions about whether certain types of injuries are underdiagnosed, or if this is an accurate number of injuries not possible to categorize more specifically. As mentioned above, maltreatment is most likely more often detected in children with psychiatric diagnoses, considering the contact with psychiatric health care. Moreover, many cases of child maltreatment are likely to go undetected, even if health care visits have been paid [45].

### Implications

Information on the increased risk for injuries in children and adolescents with certain psychiatric disorders is needed to increase the awareness among parents and caregivers. Individuals with psychiatric disorder should get access to adequate treatment and support without delay in order to reduce symptoms, which in turn, could have an effect on injury prevalence. Finally, more than one out of three children and adolescents were diagnosed with injuries during a five-year period, indicating that environmental and legislative prevention strategies could reduce both individual suffering and societal costs. Longitudinal studies are suggested for future research, in order to evaluate the effects of treatment as well as the direction of the relationship between for example internalizing problems and injuries.

## Conclusions

The results of the present study show a general pattern of an increased risk for concurrent injuries in children and adolescents with psychiatric diagnoses, but the associations vary by age and type of psychiatric disorder. Individuals with ASD were at lower risk for injuries requiring health care visits. Thus, disorder-specific patterns are at play in the association between psychiatric disorders and injuries; knowledge that can be of use in health care as well as school and other societal settings. Moreover, children and adolescents with many types of psychiatric disorders were at increased risk for a concurrent maltreatment diagnosis. The results add to the literature on risk factors for injuries in children and adolescents, supporting diagnosis specific patterns. Some diagnoses were associated with a marked increase in injury risk, indicating a high burden of disease for affected individuals.

## Supplementary information

**Additional file 1.** Group differences in injuries and external causes between children with different psychiatric disorders and those without during the study period 2014–2018. Crosstabs with Chi square.

## Data Availability

Data was obtained from the health care database Vega, hold by region Västra Götaland. Ethical Review Board approval was obtained for public sharing and presentation of data on group level only. This means that the data used in this study can only be used for the approved research and cannot be shared by the authors.

## Abbreviations

ADHD: Attention Deficit Hyperactivity Disorder
ASD: Autism Spectrum Disorder
CD: Conduct Disorder
CI: Confidence Interval
ICD-10: International Classification of Disease 10th revision
NSSI: Non-Suicidal Self Injury
MPH: Methylphenidate
ODD: Oppositional Defiant Disorder
OR: Odds Ratio
SES: Socioeconomic Status
