# Supplementary material for: Injuries in children and adolescents with psychiatric disorders
Source: BMC Public Health. 2020 Aug 24;20:1273. doi: 10.1186/s12889-020-09283-3 (PMC7445910; doi:10.1186/s12889-020-09283-3)
Supplement: Supplementary file 1 — Additional file 1. Group differences in injuries and external causes between children with different psychiatric disorders and those without during the study period 2014–2018. Crosstabs with Chi square. [file 12889_2020_9283_MOESM1_ESM.docx]

**Additional file**

**Group differences in injuries and external causes between children with any psychiatric disorder and those without during the study period 2014-2018. Crosstabs with Chi square.**

**Age 0-17**

| Injury type | No Psychiatric (n=319018) | Psychiatric (n=40579) | p |
| --- | --- | --- | --- |
| Injury (any) | 30.6% (n=97610) | 33.9% (n=13758) | <0.001 |
| Fracture | 8.7% (n=27729) | 10.8% (n=4378) | <0.001 |
| Wounds | 10.5% (n=33456) | 11.3% (n=4598) | <0.001 |
| Concussion | 1.9% (n=6014) | 2.9% (n=1197) | <0.001 |
| Poisoning | 0.3% (n=836) | 1.1% (n=427) | <0.001 |
| Non-specific | 14.0% (n=44563) | 16.5% (n=6712) | <0.001 |
| Maltreatment | 0.2% (n=624) | 1.5% (n=589) | <0.001 |
| Burns and corrosions | 1.5% (n=4822) | 1.3% (n=515) | <0.001 |
| Foreign body | 2.3% (n=7492) | 2.3% (n=947) | NS |
| Trauma | 0.2% (n=653) | 0.3% (n=104) | 0.033 |
| Complications | 0.5% (n=1742) | 0.9% (n=352) | <0.001 |
| Nerve injury | 0.1% (n=230) | 0.1% (n=56) | <0.001 |
| Frostbite | 0.0% (n=43) | 0.0% (n=19) | <0.001 |
| Injury of the eye | 0.6% (n=2018) | 0.8% (n=324) | <0.001 |
| Toxic effects | 0.7% (n=2279) | 0.9% (n=350) | 0.001 |
| Crushing injury | 0.7% (n=2316) | 0.8% (n=324) | NS |
| Superficial injury | 13.6% (n=43299) | 18.3% (n=7434) | <0.001 |
| Muscle injury | 0.8% (n=2399) | 1.0% (n=406) | <0.001 |
| Disloc., sprain, strain | 10.0% (n=31895) | 14.1% (n=5716) | <0.001 |
|  |  |  |  |
| External causes |  |  |  |
| Fall | 13.8% (n=43955) | 16.2% (n=6568) | <0.001 |
| Traffic accidents | 1.7% (n=5504) | 3.2% (n=1294) | <0.001 |
| Poisoning | 0.1% (n=276) | 0.2% (n=81) | <0.001 |
| Transport accidents | 0.4% (n=1233) | 0.9% (n=353) | <0.001 |
| Inanimate mech. forces | 5.5% (n=17398) | 7.4% (n=3014) | <0.001 |
| Animate mech. forces | 2.3% (n=7464) | 3.3% (n=1338) | <0.001 |
| Assault | 0.0% (n=27) | 0.0% (n=12) | <0.001 |
| Venomous animals | 0.1% (n=350) | 0.1% (n=48) | NS |
| Electric current | 0.1% (n=250) | 0.1% (n=52) | 0.001 |
| Suffocation | 0.1% (n=194) | 0.1% (29) | NS |

Note: X= 5 cases or fewer.

**Group differences in injuries and external causes between children with ADHD diagnoses and those without during the study period 2014-2018. Crosstabs with Chi square.**

**Age 0-6**

| Injury type | No ADHD (n=139131) | ADHD (n=678) | p |
| --- | --- | --- | --- |
| Injury any | 30.6% (n=42516) | 39.4% (n=267) | <0.001 |
| Fracture | 5.0% (n=6981) | 7.4% (n=50) | 0.005 |
| Wounds | 12.5% (n=17440) | 18.9% (n=128) | <0.001 |
| Concussion | 1.8% (n=2453) | 3.8% (n=26) | <0.001 |
| Poisoning | X |  |  |
| Non-specific | 14.1% (n=19555) | 21.2% (n=144) | <0.001 |
| Maltreatment | X |  |  |
| Self-injury | X |  |  |
|  |  |  |  |
| External causes |  |  |  |
| Fall | 12.0% (n=16697) | 17.3% (n=117) | <0.001 |
| Traffic accidents | 0.7% (n=936) | 1.6% (n=11) | 0.003 |
| Poisoning | X |  |  |
| Transport accidents | X |  |  |

Note: X= 5 cases or fewer.

**Age 7-12**

| Injury type | No ADHD (n=110547) | ADHD (n=3950) | p |
| --- | --- | --- | --- |
| Injury any | 34.9% (n=38528) | 37.8% (n=1492) | <0.001 |
| Fracture | 11.6% (n=12853) | 12.2% (n=482) | NS |
| Wounds | 10.9% (n=12057) | 14.6% (n=576) | <0.001 |
| Concussion | 2.1% (n=2304) | 3.1% (n=121) | <0.001 |
| Poisoning | 0.2% (n=224) | 0.2% (n=8) | NS |
| Non-specific | 16.2% (n=17876) | 17.9% (n=708) | 0.003 |
| Maltreatment | 0.2% (n=264) | 0.9% (n=35) | <0.001 |
| Self-injury | 0.1% (n=117) | 0.5% (n=20) | <0.001 |
|  |  |  |  |
| External causes |  |  |  |
| Fall | 16.3% (n=18005) | 18.2% (n=718) | 0.002 |
| Traffic accidents | 2.0% (n=2266) | 3.0% (n=117) | <0.001 |
| Poisoning | X |  |  |
| Transport accidents | 0.5% (n=554) | 0.6% (n=23) | NS |

Note: X= 5 cases or fewer.

**Age 13-17**

| Injury type | No ADHD (n=99422) | ADHD (n=5869) | p |
| --- | --- | --- | --- |
| Injury any | 26.9% (n=26703) | 31.7% (n=1862) | <0.001 |
| Fracture | 11.1% (n=10993) | 12.7% (n=748) | <0.00 |
| Wounds | 7.2% (n=7160) | 11.8% (n=693) | <0.001 |
| Concussion | 2.1% (n=2105) | 3.4% (n=202) | <0.001 |
| Poisoning | 0.4% (n=363) | 2.4% (n=138) | <0.001 |
| Non-specific | 12.2% (n=12120) | 14.9% (n=872) | <0.001 |
| Maltreatment | 0.7% (n=711) | 2.4% (n=138) | <0.001 |
| Self-injury | 0.6% (n=628) | 4.2% (n=244) | <0.001 |
|  |  |  |  |
| External causes |  |  |  |
| Fall | 14.1% (n=14065) | 15.7% (921) | 0.001 |
| Traffic accidents | 3.2% (n=3166) | 5.1% (n=302) | <0.001 |
| Poisoning | 0.1% (n=94) | 0.2% (n=13) | 0.003 |
| Transport accidents | 0.9% (n=868) | 1.1% (n=66) | 0.046 |

Note: X= 5 cases or fewer.

**Age 0-17**

| Injury type | No ADHD (n=349100) | ADHD (n=10497) | p |
| --- | --- | --- | --- |
| Injury (any) | 30.9% (n=10774) | 34.5% (n=3641) | <0.001 |
| Fracture | 8.8% (n=30827) | 12.2% (n=1280) | <0.001 |
| Wounds | 10.5% (n=36657) | 13.3% (n=1397) | <0.001 |
| Concussion | 2.0% (n=6862) | 3.3% (n=349) | <0.001 |
| Poisoning | 0.3% (n=1114) | 1.4% (n=149) | <0.001 |
| Non-specific | 14.2% (n=49551) | 16.4% (n=1724) | <0.001 |
| Maltreatment | 0.3% (n=624) | 1.7% (n=589) | <0.001 |
| Self-injury | 0.2% (n=766) | 2.5% (n=267) | <0.001 |
| Burns and corrosions | 1.5% (n=5203) | 1.3% (n=134) | NS |
| Foreign body | 2.3% (n=8172) | 2.5% (n=267) | NS |
| Trauma | 0.2% (n=725) | 0.3% (n=32) | 0.032 |
| Complications | 0.6% (n=1993) | 1.0% (n=101) | <0.001 |
| Nerve injury | 0.1% (n=272) | 0.1% (n=14) | 0.047 |
| Frostbite | X |  |  |
| Injury of the eye | 0.6% (n=2259) | 0.8% (n=83) | NS |
| Toxic effects | 0.7% (n=2535) | 0.9% (n=94) | 0.045 |
| Crushing injury | 0.7% (n=2558) | 0.8% (n=82) | NS |
| Superficial injury | 13.9% (n=48641) | 19.9% (n=2092) | <0.001 |
| Muscle injury | 0.8% (n=2701) | 1.0% (n=104) | 0.013 |
| Disloc., sprain, strain | 10.3% (n=36049) | 14.9% (n=1562) | <0.001 |
|  |  |  |  |
| External causes |  |  |  |
| Fall | 14.0% (n=48767) | 16.7% (n=1756) | <0.001 |
| Traffic accidents | 1.8% (n=6368) | 4.1% (n=430) | <0.001 |
| Poisoning | 0.1% (n=338) | 0.2% (n=19) | 0.007 |
| Transport accidents | 0.4% (n=1497) | 0.8% (n=89) | <0.001 |
| Inanimate mech. forces | 5.6% (n=19483) | 8.9% (n=929) | <0.001 |
| Animate mech. forces | 2.4% (n=8386) | 4.0% (n=416) | <0.001 |
| Assault | X |  |  |
| Venomous animals | 0.1% (n=391) | 0.1% (n=7) | NS |
| Electric current | 0.1% (n=284) | 0.2% (n=18) | 0.002 |
| Suffocation | 0.1% (n=216) | 0.1% (7) | NS |

Note: X= 5 cases or fewer.

**Group differences in injuries and external causes between children with ODD and CD diagnoses and those without during the study period 2014-2018. Crosstabs with Chi square.**

**Age 0-6**

| Injury type | No ODD/CD (n=139651) | ODD/CD (n=158) | p |
| --- | --- | --- | --- |
| Injury any | 30.6% (n=42725) | 36.7% (n=58) | NS |
| Fracture | 5.0% (n=7018) | 8.2% (n=13) | NS (p=0.066) |
| Wounds | 12.6% (n=17540) | 17.7% (n=28) | NS (p=0.050) |
| Concussion | X |  |  |
| Poisoning | X |  |  |
| Non-specific | 14.1% (n=19666) | 20.9% (n=33) | 0.014 |
| Maltreatment | X |  |  |
| Self-injury | X |  |  |
|  |  |  |  |
| External causes |  |  |  |
| Fall | 12.0% (n=16787) | 17.1% (n=27) | NS (p=0.050) |
| Traffic accidents | X |  |  |
| Poisoning | X |  |  |
| Transport accidents | X |  |  |

Note: X= five cases or fewer.

**Age 7-12**

| Injury type | No ODD/CD (n=113482) | ODD/CD (n=655) | p |
| --- | --- | --- | --- |
| Injury any | 34.9% (n=39746) | 41.8% (n=274) | <0.001 |
| Fracture | 11.6% (n=13237) | 15.0% (n=98) | 0.008 |
| Wounds | 11.0% (n=12537) | 14.7% (n=96) | 0.003 |
| Concussion | 2.1% (n=2400) | 3.8% (n=25) | 0.002 |
| Poisoning | X |  |  |
| Non-specific | 16.6% (n=18473) | 16.9% (n=111) | NS |
| Maltreatment | 0.3% (n=290) | 1.4% (n=9) | <0.001 |
| Self-injury | 0.1% (n=127) | 1.5% (n=10) | <0.001 |
|  |  |  |  |
| External causes |  |  |  |
| Fall | 16.3% (n=118579) | 22.0% (n=144) | <0.001 |
| Traffic accidents | 2.1% (n=2356) | 4.1% (n=27) | 0.001 |
| Poisoning | X |  |  |
| Transport accidents | X |  |  |

Note: X= five cases or fewer.

**Age 13-17**

| Injury type | No ODD/CD (n=104952) | ODD/CD (n=339) | p |
| --- | --- | --- | --- |
| Injury any | 27.1% (28411) | 45.4% (n=154) | <0.001 |
| Fracture | 11.1% (n=11673) | 20.1% (n=68) | <0.001 |
| Wounds | 7.4% (n=7800) | 15.6% (n=53) | <0.001 |
| Concussion | 2.2% (n=2286) | 6.2% (n=21) | <0.001 |
| Poisoning | 0.5% (n=493) | 2.4% (n=8) | <0.001 |
| Non-specific | 12.3% (n=12934) | 17.1% (n=58) | 0.007 |
| Maltreatment | 0.8% (n=836) | 3.8% (n=13) | <0.001 |
| Self-injury | 0.8% (n=847) | 7.4% (n=25) | <0.001 |
|  |  |  |  |
| External causes |  |  |  |
| Fall | 14.2% (n=14902) | 24.8% (n=84) | <0.001 |
| Traffic accidents | 3.3% (n=3441) | 8.0% (n=27) | <0.001 |
| Poisoning | X |  |  |
| Transport accidents | X |  |  |

Note: X= five cases or fewer.

**Age 0-17**

| Injury type | No ODD/CD (n=358445) | CD/ODD (n=1152) | p |
| --- | --- | --- | --- |
| Injury (any) | 30.9% (n=110882) | 42.2% (n=486) | <0.001 |
| Fracture | 8.9% (n=31928) | 15.5% (n=179) | <0.001 |
| Wounds | 10.6% (n=37877) | 15.4% (n=177) | <0.001 |
| Concussion | 2.0% (n=7163) | 4.2% (n=48) | <0.001 |
| Poisoning | 0.3% (n=1248) | 1.3% (n=15) | <0.001 |
| Non-specific | 14.2% (n=51073) | 17.5% (n=202) | <0.001 |
| Maltreatment | 0.3% (n=1190) | 2.0% (n=23) | <0.001 |
| Self-injury | 0.3% (n=996) | 3.2% (n=37) | <0.001 |
| Burns and corrosions | 1.5% (n=5323) | 1.2% (n=14) | NS |
| Foreign body | 2.3% (n=8408) | 2.7% (n=31) | NS |
| Trauma | X |  |  |
| Complications | 0.6% (n=2086) | 0.7% (n=8) | NS |
| Nerve injury | X |  |  |
| Frostbite | X |  |  |
| Injury of the eye | 0.7% (n=2335) | 0.6% (n=7) | NS |
| Toxic effects | 0.7% (n=2620) | 0.8% (n=9) | NS |
| Crushing injury | 0.7% (n=2625) | 1.3% (n=15) | 0.024 |
| Superficial injury | 14.1% (n=50451) | 24.5% (n=282) | <0.001 |
| Muscle injury | 0.8% (n=2789) | 1.4% (n=16) | 0.019 |
| Disloc., sprain, strain | 10.4% (n=37422) | 16.4% (n=189) | <0.001 |
|  |  |  |  |
| External causes |  |  |  |
| Fall | 14.0% (n=50268) | 22.1% (n=255) | <0.001 |
| Traffic accidents | 1.9% (n=6742) | 4.9% (n=56) | <0.001 |
| Poisoning | X |  |  |
| Transport accidents | 0.4% (n=1575) | 1.0% (n=11) | 0.008 |
| Inanimate mech. forces | 5.7% (n=20288) | 10.8% (n=124) | <0.001 |
| Animate mech. forces | 2.4% (n=8751) | 4.4% (n=51) | <0.001 |
| Assault | X |  |  |
| Venomous animals | X |  |  |
| Electric current | X |  |  |
| Suffocation | X |  |  |

Note: X= 5 cases or fewer.

**Group differences in injuries and external causes between children with anxiety diagnoses and those without during the study period 2014-2018. Crosstabs with Chi square.**

**Age 0-6**

| Injury type | No anxiety (n=139314) | Anxiety (n=184) | p |
| --- | --- | --- | --- |
| Injury any | 30.6% (n=42599) | 37.2% (n=184) | 0.001 |
| Fracture | 5.0% (n=6992) | 7.9% (n=39) | 0.004 |
| Wounds | 12.6% (n=17506) | 12.5% (n=62) | NS |
| Concussion | 1.8% (n=2467) | 2.4% (n=12) | NS |
| Poisoning | X |  |  |
| Non-specific | 14.1% (n=19592) | 21.6% (n=107) | <0.001 |
| Maltreatment | 0.0% (n=58) | 1.4% (n=7) | <0.001 |
| Self-injury | X |  |  |
|  |  |  |  |
| External causes |  |  |  |
| Fall | 12.0% (n=16745) | 13.9% (n=69) | NS |
| Traffic accidents | 0.7% (n=937) | 2.0% (n=10) | <0.001 |
| Poisoning | X |  |  |
| Transport accidents | X |  |  |

Note: X= five cases or fewer.

**Age 7-12**

| Injury type | No anxiety (n=109329) | Anxiety (n=5168) | p |
| --- | --- | --- | --- |
| Injury any | 34.6% (n=37863) | 41.7% (n=2157) | <0.001 |
| Fracture | 11.5% (n=12624) | 13.8% (n=711) | <0.001 |
| Wounds | 11.0% (n=12077) | 10.8% (n=556) | NS |
| Concussion | 2.0% (n=2237) | 3.6% (n=188) | <0.001 |
| Poisoning | 0.2% (n=209) | 0.4% (n=23) | <0.001 |
| Non-specific | 16.0% (n=17510) | 20.8% (n=1074) | <0.001 |
| Maltreatment | 0.2% (n=209) | 1.7% (n=90) | <0.001 |
| Self-injury | 0.0% (n=48) | 1.7% (n=89) | <0.001 |
|  |  |  |  |
| External causes |  |  |  |
| Fall | 16.2% (n=17668) | 20.4% (n=1055) | <0.001 |
| Traffic accidents | 2.0% (n=2226) | 3.0% (n=157) | <0.001 |
| Poisoning | X |  |  |
| Transport accidents | 0.5% (n=518) | 1.1% (n=59) | <0.001 |

Note: X=five cases or fever

**Age 13-17**

| Injury type | No anxiety (n=93505) | Anxiety (n=11786) | p |
| --- | --- | --- | --- |
| Injury any | 26.7% (n=24966) | 30.5% (n=3599) | <0.001 |
| Fracture | 11.3% (n=10529) | 10.3% (n=1212) | 0.001 |
| Wounds | 7.3% (n=6865) | 8.4% (n=988) | <0.001 |
| Concussion | 2.0% (n=1881) | 3.6% (n=426) | <0.001 |
| Poisoning | 0.2% (n=219) | 2.4% (n=282) | <0.001 |
| Non-specific | 12.0% (n=11205) | 15.2% (n=1787) | <0.001 |
| Maltreatment | 0.6% (n=515) | 2.8% (n=334) | <0.001 |
| Self-injury | 0.2% (n=183) | 5.8% (n=689) | <0.001 |
|  |  |  |  |
| External causes |  |  |  |
| Fall | 14.1% (n=13195) | 15.2% (n=1791) | 0.001 |
| Traffic accidents | 3.2% (n=2997) | 4.0% (n=471) | <0.001 |
| Poisoning | 0.1% (n=78) | 0.2% (n=29) | <0.001 |
| Transport accidents | 0.8% (n=760) | 1.5% (n=174) | <0.001 |

Note: X= five cases or fewer.

**Age 0-17**

| Injury type | No Anxiety (n=342148) | Anxiety (n=17449) | p |
| --- | --- | --- | --- |
| Injury (any) | 30.8% (n=105428) | 34.0% (n=5904) | <0.001 |
| Fracture | 8.8% (n=30145) | 11.2% (n=1962) | <0.001 |
| Wounds | 10.7% (n=36448) | 9.2% (n=1606) | <0.001 |
| Concussion | 1.9% (n=6585) | 3.6% (n=626) | <0.001 |
| Poisoning | 0.3% (n=957) | 1.8% (n=306) | <0.001 |
| Non-specific | 14.1% (n=48307) | 17.0% (n=2968) | <0.001 |
| Maltreatment | 0.2% (n=782) | 2.5% (n=431) | <0.001 |
| Self-injury | 0.1% (n=252) | 4.5% (n=781) | <0.001 |
| Burns and corrosions | 1.5% (n=5139) | 1.1% (n=198) | <0.001 |
| Foreign body | 2.4% (n=8118) | 1.8% (n=321) | <0.001 |
| Trauma | 0.2% (n=708) | 0.3% (n=49) | 0.038 |
| Complications | 0.6% (n=1936) | 0.9% (n=158) | <0.001 |
| Nerve injury | 0.1% (n=254) | 0.2% (n=32) | <0.001 |
| Frostbite | 0.0% (n=51) | 0.1% (n=11) | <0.001 |
| Injury of the eye | 0.6% (n=2212) | 0.7% (n=130) | NS |
| Toxic effects | 0.7% (n=2472) | 0.9% (n=157) | 0.007 |
| Crushing injury | 0.7% (n=2522) | 0.7% (n=118) | NS |
| Superficial injury | 13.8% (n=47315) | 19.6% (n=3418) | <0.001 |
| Muscle injury | 0.8% (n=2573) | 1.3% (n=232) | <0.001 |
| Disloc., sprain, strain | 10.1% (n=34641) | 17.0% (n=2970) | <0.001 |
|  |  |  |  |
| External causes |  |  |  |
| Fall | 13.9% (n=47608) | 16.7% (n=2915) | <0.001 |
| Traffic accidents | 1.8% (n=6160) | 3.7% (n=638) | <0.001 |
| Poisoning | 0.1% (n=322) | 0.2% (n=35) | <0.001 |
| Transport accidents | 0.4% (n=1352) | 1.3% (n=234) | <0.001 |
| Inanimate mech. forces | 5.6% (n=19109) | 7.5% (n=1303) | <0.001 |
| Animate mech. forces | 2.4% (n=8109) | 4.0% (n=693) | <0.001 |
| Assault | 0.0% (n=30) | 0.1% (n=9) | <0.001 |
| Venomous animals | 0.1% (n=375) | 0.1% (n=23) | NS |
| Electric current | 0.1% (n=283) | 0.1% (n=19) | NS |
| Suffocation | 0.1% (n=211) | 0.1% (n=12) | NS |

Note: X= 5 cases or fewer.

**Group differences in injuries and external causes between children with autism spectrum diagnoses and those without during the study period 2014-2018. Crosstabs with Chi square.**

**Age 0-6**

| Injury type | No Autism (n=139012) | Autism (n=797) | p |
| --- | --- | --- | --- |
| Injury any | 30.6% (n=42540) | 30.5% (n=243) | NS |
| Fracture | 5.0% (n=6982) | 6.1% (n=49) | NS |
| Wounds | 12.6% (n=17463) | 13.2% (n=105) | NS |
| Concussion | 1.8% (n=2457) | 2.8% (n=22) | 0.034 |
| Poisoning | X |  |  |
| Non-specific | 14.1% (n=19559) | 17.6% (n=140) | 0.005 |
| Maltreatment | X |  |  |
| Self-injury | X |  |  |
|  |  |  |  |
| External causes |  |  |  |
| Fall | 12.0% (n=16714) | 12.5% (n=100) | NS |
| Traffic accidents | X |  |  |
| Poisoning | X |  |  |
| Transport accidents | X |  |  |

Note: X= five cases or fewer.

**Age 7-12**

| Injury type | No Autism (n=112851) | Autism (n=1646) | p |
| --- | --- | --- | --- |
| Injury any | 35.0% (n=39459) | 30.8% (n=561) | <0.001 |
| Fracture | 11.7% (n=13147) | 10.3% (n=188) | NS |
| Wounds | 11.0% (n=12433) | 11.0% (n=200) | NS |
| Concussion | 2.1% (n=2386) | 2.1% (n=39) | NS |
| Poisoning | X |  |  |
| Non-specific | 16.2% (n=18283) | 16.5% (n=301) | NS |
| Maltreatment | 0.3% (n=287) | 0.7% (n=12) | 0.001 |
| Self-injury | 0.1% (n=125) | 0.7% (n=12) | <0.001 |
|  |  |  |  |
| External causes |  |  |  |
| Fall | 16.4% (n=18456) | 14.7% (n=267) | 0.049 |
| Traffic accidents | 2.1% (n=2347) | 2.0% (n=36) | NS |
| Poisoning | X |  |  |
| Transport accidents | 0.5% (n=571) | 0.3% (n=6) | NS |

Note: X= five cases or fewer.

**Age 13-17**

| Injury type | No Autism (n=102830) | Autism (n=2461) | p |
| --- | --- | --- | --- |
| Injury any | 27.2% (n=27993) | 23.2% (n=572) | <0.001 |
| Fracture | 11.2% (n=11527) | 8.7% (n=214) | <0.001 |
| Wounds | 7.4% (n=7652) | 8.2% (n=201) | NS |
| Concussion | 2.2% (n=2256) | 2.1% (n=51) | NS |
| Poisoning | 0.4% (n=442) | 2.4% (n=59) | <0.001 |
| Non-specific | 12.4% (n=12708) | 11.5% (n=284) | NS |
| Maltreatment | 0.8% (n=813) | 1.5% (n=36) | <0.001 |
| Self-injury | 0.7% (n=768) | 4.2% (n=104) | <0.001 |
|  |  |  |  |
| External causes |  |  |  |
| Fall | 14.3% (n=14736) | 10.2% (n=250) | <0.001 |
| Traffic accidents | 3.3% (n=3396) | 2.9% (n=72) | NS |
| Poisoning | X |  |  |
| Transport accidents | 0.9% (n=919) | 0.6% (n=15) | NS |

Note: X= five cases or fewer.

**Age 0-17**

| Injury type | No Autism (n=354518) | Autism (n=5079) | p |
| --- | --- | --- | --- |
| Injury (any) | 31.0% (n=110882) | 27.1% (n=1376) | <0.001 |
| Fracture | 8.9% (n=31656) | 8.9% (n=451) | NS |
| Wounds | 10.6% (n=37548) | 10.0% (n=506) | NS |
| Concussion | 2.0% (n=7099) | 2.2% (n=112) | NS |
| Poisoning | 0.3% (n=1198) | 1.3% (n=65) | <0.001 |
| Non-specific | 14.3% (n=50550) | 14.3% (n=725) | NS |
| Maltreatment | 0.3% (n=1162) | 1.0% (n=51) | <0.001 |
| Self-injury | 0.3% (n=917) | 2.3% (n=116) | <0.001 |
| Burns and corrosions | 1.5% (n=5276) | 1.2% (n=61) | NS |
| Foreign body | 2.3% (n=8300) | 2.7% (n=139) | NS |
| Trauma | 0.2% (n=744) | 0.3% (n=13) | NS |
| Complications | 0.6% (n=2037) | 1.1% (n=57) | <0.001 |
| Nerve injury | X |  |  |
| Frostbite | X |  |  |
| Injury of the eye | 0.7% (n=2314) | 0.6% (n=28) | NS |
| Toxic effects | 0.7% (n=2589) | 0.8% (n=40) | NS |
| Crushing injury | 0.7% (n=2598) | 0.8% (n=42) | NS |
| Superficial injury | 14.1% (n=50026) | 13.9% (n=707) | NS |
| Muscle injury | 0.8% (n=2779) | 0.5% (n=26) | 0.029 |
| Disloc., sprain, strain | 10.5% (n=37124) | 9.6% (n=487) | 0.041 |
|  |  |  |  |
| External causes |  |  |  |
| Fall | 14.1% (n=49906) | 12.1% (n=617) | <0.001 |
| Traffic accidents | 1.9% (n=6687) | 2.2% (n=111) | NS |
| Poisoning | 0.1% (n=349) | 0.2% (n=8) | NS |
| Transport accidents | 0.4% (n=1561) | 0.5% (n=25) | NS |
| Inanimate mech. forces | 5.7% (n=20109) | 6.0% (n=303) | NS |
| Animate mech. forces | 2.4% (n=8670) | 2.6% (n=132) | NS |
| Assault | X |  | NS |
| Venomous animals | X |  |  |
| Electric current | 0.1% (n=294) | 0.2% (n=8) | NS |
| Suffocation | X |  |  |

Note: X= 5 cases or fewer.

**Group differences in injuries and external causes between children with Affective disorders and those without during the study period 2014-2018. Crosstabs with Chi square.**

**Age 0-6**

| Injury type | No Affective (n=139786) | Affective (n=23) | p |
| --- | --- | --- | --- |
| Injury any | 30.6% (n=42777) | 26.1% (n=6) | NS |
| Fracture | X |  |  |
| Wounds | X |  |  |
| Concussion | X |  |  |
| Poisoning | X |  |  |
| Non-specific | X |  |  |
| Maltreatment | X |  |  |
| Self-injury | X |  |  |
|  |  |  |  |
| External causes |  |  |  |
| Fall | X |  |  |
| Traffic accidents | X |  |  |
| Poisoning | X |  |  |
| Transport accidents | X |  |  |

Note: X= five cases or fewer.

**Age 7-12**

| Injury type | No Affective (n=113346) | Affective (n=1151) | p |
| --- | --- | --- | --- |
| Injury any | 34.9% (n=39539) | 41.8% (n=481) | <0.001 |
| Fracture | 11.6% (n=13167) | 14.6% (n=168) | 0.002 |
| Wounds | 11.0% (n=12502) | 11.4% (n=131) | NS |
| Concussion | 2.1% (n=2374) | 4.4% (n=51) | <0.001 |
| Poisoning | 0.2% (n=224) | 0.7% (n=8) | <0.001 |
| Non-specific | 16.2% (n=18360) | 19.5% (n=224) | 0.003 |
| Maltreatment | 0.3% (n=289) | 0.9% (n=10) | <0.001 |
| Self-injury | 0.1% (n=83) | 4.7% (n=54) | <0.001 |
|  |  |  |  |
| External causes |  |  |  |
| Fall | 16.3% (n=18461) | 22.8% (n=262) | <0.001 |
| Traffic accidents | 2.1% (n=2347) | 3.1% (n=36) | 0.012 |
| Poisoning | X |  |  |
| Transport accidents | 0.5% (n=566) | 1.0% (n=11) | 0.030 |

Note: X= five cases or fewer.

**Age 13-17**

| Injury type | No Affective (n=98905) | Affective (n=6386) | p |
| --- | --- | --- | --- |
| Injury any | 27.0% (n=26699) | 29.2% (n=1866) | <0.001 |
| Fracture | 11.3% (n=11189) | 8.6% (n=552) | <0.001 |
| Wounds | 7.3% (n=7259) | 9.3% (n=594) | <0.001 |
| Concussion | 2.1% (n=2080) | 3.6% (n=227) | <0.001 |
| Poisoning | 0.3% (n=253) | 3.9% (n=248) | <0.001 |
| Non-specific | 12.2% (n=12070) | 14.4% (n=922) | <0.001 |
| Maltreatment | 0.7% (n=675) | 2.7% (n=174) | <0.001 |
| Self-injury | 0.3% (n=302) | 8.9% (n=570) | <0.001 |
|  |  |  |  |
| External causes |  |  |  |
| Fall | 14.2% (n=14092) | 14.0% (n=894) | NS |
| Traffic accidents | 3.2% (n=13199) | 4.2% (n=269) | <0.001 |
| Poisoning | 0.1% (n=92) | 0.2% (n=15) | 0.001 |
| Transport accidents | 0.8% (n=840) | 1.5% (n=94) | <0.001 |

Note: X= five cases or fewer.

**Age 0-17**

| Injury type | No Affective (n=352037) | Affective (n=7560) | p |
| --- | --- | --- | --- |
| Injury (any) | 31.0% (n=109015) | 31.1% (n=2353) | NS |
| Fracture | 8.9% (n=31384) | 9.6% (n=723) | NS (p=0.050) |
| Wounds | 10.6% (n=37326) | 9.6% (n=728) | 0.006 |
| Concussion | 2.0% (n=6399) | 3.7% (n=278) | <0.001 |
| Poisoning | 0.3% (n=1007) | 3.4% (n=256) | <0.001 |
| Non-specific | 14.2% (n=50128) | 15.2% (n=1147) | 0.022 |
| Maltreatment | 0.3% (n=1029) | 2.4% (n=184) | <0.001 |
| Self-injury | 0.1% (n=408) | 8.3% (n=625) | <0.001 |
| Burns and corrosions | 1.5% (n=5270) | 0.9% (n=67) | <0.001 |
| Foreign body | 2.4% (n=8326) | 1.5% (n=113) | <0.001 |
| Trauma | 0.2% (n=734) | 0.3% (n=23) | NS |
| Complications | 0.6% (n=2013) | 1.1% (n=81) | <0.001 |
| Nerve injury | 0.1% (n=268) | 0.2% (n=18) | <0.001 |
| Frostbite | X |  |  |
| Injury of the eye | 0.7% (n=2294) | 0.6% (n=48) | NS |
| Toxic effects | 0.7% (n=2561) | 0.9% (n=68) | NS |
| Crushing injury | 0.7% (n=2598) | 0.6% (n=42) | NS |
| Superficial injury | 14.0% (n=49315) | 18.8% (n=1418) | <0.001 |
| Muscle injury | 0.8% (n=2711) | 1.2% (n=94) | <0.001 |
| Disloc., sprain, strain | 10.3% (n=36363) | 16.5% (n=1248) | <0.001 |
|  |  |  |  |
| External causes |  |  |  |
| Fall | 14.0% (n=49364) | 15.3% (n=1159) | 0.001 |
| Traffic accidents | 1.8% (n=6492) | 4.0% (n=306) | <0.001 |
| Poisoning | 0.1% (n=341) | 0.2% (n=16) | <0.002 |
| Transport accidents | 0.4% (n=1481) | 1.4% (n=105) | <0.001 |
| Inanimate mech. forces | 5.6% (n=19873) | 7.1% (n=539) | <0.001 |
| Animate mech. forces | 2.4% (n=8489) | 4.1% (n=313) | <0.001 |
| Assault | X |  | NS |
| Venomous animals | 0.1% (n=390) | 0.1% (n=8) | NS |
| Electric current | 0.1% (n=295) | 0.1% (n=7) | NS |
| Suffocation | 0.1% (n=215) | 0.1% (n=8) | NS |

Note: X= 5 cases or fewer.

**Group differences in injuries and external causes between children with Psychotic disorders and those without during the study period 2014-2018. Crosstabs with Chi square.**

**Age 0-17**

| Injury type | No Psychosis (n=359481) | Psychosis (n=116) | p |
| --- | --- | --- | --- |
| Injuries (any) | 31.0% (n=111336) | 27.6% (n=32) | NS |
| Fracture | 8.9% (n=32092) | 12.9% (n=15) | NS |
| Wounds | 10.6% (n=38043) | 9.5% (n=11) | NS |
| Concussion | X |  |  |
| Poisoning | 0.3% (n=1256) | 6.0% (n=7) | <0.001 |
| Non-specific | 14.3% (n=51260) | 12.9% (n=15) | NS |
| Maltreatment | X |  |  |
| Self-injury | 0.3% (n=1020) | 11.2% (n=13) | <0.001 |
| Burns and corrosions | X |  |  |
| Foreign body | 2.3% (n=8433) | 5.2% (n=6) | 0.044 |
| Trauma | X |  |  |
| Complications | X |  |  |
|  |  |  |  |
| External causes |  |  |  |
| Fall | 14.0% (n=50503) | 17.2% (n=20) | NS |
| Traffic accidents | X |  |  |
| Poisoning | X |  |  |
| Transport accidents | X |  |  |
| Inanimate mech forces | 5.7% (n=20403) | 7.8% (n=9) | NS |
| Animate mech forces | X |  |  |
| Assault | X |  |  |
| Venomous animals | X |  |  |
| Electric current | X |  |  |
| Suffocation | X |  |  |

Note: X= five cases or fewer.
